# Hyponatremia from oxcarbazepine: A case report

**DOI:** 10.1192/j.eurpsy.2022.432

**Published:** 2022-09-01

**Authors:** P. Coucheiro Limeres, A. Cerame, S. Maldonado Orellana, A. Franco Soler

**Affiliations:** Hospital Universitario José Germain, Psychiatry Department, Leganés, Spain

**Keywords:** hyponatremia, Schizoaffective disorder, oxcarbazepine

## Abstract

**Introduction:**

Oxcarbazepine (OXC) is an antiepileptic drug used as a mood stabilizer in patients diagnosed with bipolar disorder (BD). OXC has been reported as a source of hyponatremia in its use in both epilepsy and BD.

**Objectives:**

We present the case of a 53 year-old male patient diagnosed with Schizoaffective disorder, bipolar type who developed hyponatremia during his treatment with OXC.

**Methods:**

The patient’s treatment was desvelafaxine 100 mg, Paliperidone depot 150 mg, Oxcarbazepine 600 mg which he had maintained for at least one year. He began to manifest headache, asthenia and mild confusion gradually, with morning predominance, without being clearly suggestive of an acute worsening.

**Results:**

In control analysis, the existence of sodium leveles of 127 and low osmolarity was observed. Therefore it was decided to suspend furosemide, close monitoring of water intake in order to rule out primary polydipsia and extra salt was introduced into the diet. Given the persistence of the symptoms, laboratory abnormalities and ruled out the existence of primary polydipsia, it was decided to suspend treatment with oxcarbazepine. After the discontinuation of the aforementioned drug the analytical findings went back to normal ranges and the symptoms disappeared.

**Conclusions:**

Carrying out control tests in patients with psychiatric pathology and multiple psychiatric treatments is essential to be able to rule out analytical alterations which could be asymptomatic or with nonspecific symptoms that could be attributed to the underlying pathology. The easy reversal of symptoms encourages us to emphasize the study and differential diagnosis of each case.

**Disclosure:**

No significant relationships.

